# Physical Therapy in Palliative Care: From Symptom Control to Quality of Life: A Critical Review

**DOI:** 10.4103/0973-1075.73670

**Published:** 2010

**Authors:** Senthil P Kumar, Anand Jim

**Affiliations:** Department of Physiotherapy, Kasturba Medical College, Manipal University, Mangalore, India; 1 Department of Physiotherapy, Bethany Navjeevan College of Physiotherapy, Trivandrum, India

**Keywords:** Palliative physiotherapy, Quality of life, Rehabilitation, Therapeutic modalities

## Abstract

Physiotherapy is concerned with identifying and maximizing movement potential, within the spheres of promotion, prevention, treatment and rehabilitation. Physical therapists practice in a broad range of inpatient, outpatient, and community-based settings such as hospice and palliative care centers where as part of a multidisciplinary team of care, they address the physical and functional dimensions of the patients’ suffering. Physiotherapy treatment methods like therapeutic exercise, electrical modalities, thermal modalities, actinotherapy, mechanical modalities, manual physical therapy and assistive devices are useful for a range of life-threatening and life-limiting conditions like cancer and cancer-associated conditions; HIV; neurodegenerative disorders like amyotrophic lateral sclerosis, multiple sclerosis; respiratory disorders like idiopathic pulmonary fibrosis; and altered mental states. The professional armamentarium is still expanding with inclusion of other miscellaneous techniques which were also proven to be effective in improving quality of life in these patients. Considering the scope of physiotherapy in India, and in palliative care, professionals in a multidisciplinary palliative care team need to understand and mutually involve toward policy changes to successfully implement physical therapeutic palliative care delivery.

## INTRODUCTION

World Confederation for Physical Therapy (WCPT) defines Physical Therapy as; “… providing services to people and populations to develop, maintain and restore maximum movement and functional ability throughout the life-span. Physiotherapy includes the provision of services in circumstances where movement and function are threatened by the process of ageing or that of injury or disease. Full and functional movement are at the heart of what it means to be healthy. Physiotherapy is concerned with identifying and maximizing movement potential, within the spheres of promotion, prevention, treatment and rehabilitation. Physiotherapy involves the interaction between physiotherapist, patients or clients, families and care givers, in a process of assessing movement potential and in establishing agreed upon goals and objectives using knowledge and skills unique to physiotherapists”.[1]

Physical therapists practice in a broad range of inpatient, outpatient, and community-based settings, including the following:[2]

- Hospitals (eg, critical care, intensive care, acute care, and subacute care settings)
- Outpatient clinics or offices
- Rehabilitation facilities
- Skilled nursing, extended care, or subacute facilities
- Homes
- Education or research centers
- Schools and playgrounds (preschool, primary, and secondary)
- Hospices or palliative care centers
- Corporate or industrial health centers
- Industrial, workplace, or other occupational environments
- Athletic facilities (collegiate, amateur, and professional)
- Fitness centers and sports training facilities.

Physiotherapists play an inherent role in the multidisciplinary palliative care team emphasizing on improving function and quality of life in patients who are deemed to require physical and functional dimensions of care.[3] Physical dimension was defined as one’s experience of the physical discomfort associated with progressive illness for a perceived level of physical distress.[4] Physical dimension in physical therapy includes symptom control, management of physical findings such as mobility, strength, flexibility, endurance, deformity, co-ordination, balance, gait, breathing, exercise tolerance and energy expenditure.[5] Symptom control by physical therapy is applicable in patients with commonest symptoms which require palliative care such as pain, weakness, cough and breathlessness.[6]

Functional dimension is defined as one’s perceived ability to perform accustomed functions and activities of daily living, experienced in relation to expectations and adaptations to declining functionality.[3] Functional limitations include sensorimotor performance in the execution of particular actions, tasks, and activities (eg, rolling, getting out of bed, transferring, walking, climbing, bending, lifting, carrying). These sensorimotor functional abilities underlie the daily, fundamental organized patterns of behaviors that are classified as basic activities of daily living (ADL) (eg, feeding, dressing, bathing, grooming, toileting). The more complex tasks associated with independent community living (eg, use of public transportation, grocery shopping) are categorized as instrumental activities of daily living (IADL). Successful performance of complex physical functional activities, such as personal hygiene and housekeeping, typically requires integration of cognitive and affective abilities as well as physical ones.[7]

Physicians address the physical dimension to their extent and nurses in functional dimension. Addressing both the aspects simultaneously can be more beneficial for the patient. Considering the current healthcare scenario in India as reported by Seamark *et al*.[8] *Inspite of most of population being rural, the current status of medical personnel and facilities in our country is scarce; with only 34% of physicians and 25% of all hospital beds are available in rural areas. Nursing is considered as a low-status job and is not a much-sought-after profession for young people*. Hence the need for an efficient allied health professional to fill in the current needs of palliative care team sees the emergence of physical therapists, with their thorough professional background and clinical expertise, as invaluable members in the team of care.

The objective of this paper is to update the palliative care clinicians, physical therapists and other team members on the role of a physical therapist in a palliative care team by detailed view of physical therapy treatment methods and their evidence for application into conditions requiring palliative care.

## PHYSICAL THERAPY TECHNIQUES

### Therapeutic exercise

It comprises passive movement, assisted active movement, active movement, assisted-resisted active movement, and resisted movement techniques. The techniques can be applied in anatomical planes or as functional movement direction. The techniques can be performed on land or in water. The latter is termed as “hydrotherapy”. Best examples of therapeutic exercise techniques are relaxation, massage, suspension therapy, muscle- re-education, progressive resisted exercise, floor aerobics, active mobility exercises, mobilization and stabilization exercise, proprioceptive neuromuscular facilitation (facilitation and inhibition techniques); breathing exercise; postural training; work simulation, work conditioning and work hardening; graded activity program and cognitive-behavioral training.[2] Exercises are useful for reconditioning and physical fitness.

### Electrical modalities

Low-frequency modalities like neuromuscular electrical stimulation (both galvanic and faradic) and functional electrical stimulation, iontophoresis, high-voltage pulsed galvanic current, transcutaneous electrical nerve stimulation (TENS) and diadynamic currents; medium frequency modalities like Russian currents and Interferential therapy. High frequency modalities are usually grouped under deep-heating modalities under thermal modalities. Electrical modalities are very useful adjuncts in pain management.

### Thermal modalities

Cryotherapy (ice massage, cold pack, cold bath, vapocoolant spray); superficial heating agents (fluidotherapy, hot pack, infrared radiation, paraffin wax; and contrast baths. Deep heating agents (diathermy- shortwave and microwave, ultrasound and phonophoresis); hydrotherapy (whirlpool and contrast bath). Thermal modalities are effective adjuncts to exercise and/or electrical modalities.

### Mechanical modalities

Traction therapy, compression therapy, therapeutic taping and continuous passive motion. Compression therapy can be very useful in managing lymphedema.

### Additional physical agents- Actinotherapy

Ultraviolet, LASER, Extracorporeal Shock Wave therapy are useful in selected situations.

### Miscellaneous modalities

Biofeedback is useful in patients with limited cognitive abilities and neuromuscular dyscontrol.

### Manual physical therapy (manual therapy)

#### Myofascial

Massage, deep transverse frictions, myofascial release, trigger point therapy, muscle energy techniques, motor control retraining.

#### Articular

Joint mobilization using passive physiological and passive accessory (joint play) movements, combined movements, mobilization with movements, manipulation (high-velocity low amplitude thrust techniques).

#### Neural

Neurodynamic techniques of neural tissue loading and nerve massage.

Manual physical therapy techniques are used in a variety of settings ranging from hospital-based to home-based. The effects of the techniques depend upon the skills of the handling therapist.

#### Assistive devices

Orthosis- splints/ braces: supportive devices for the body parts.

Prosthesis: artificial limbs.

Mobility aids: locomotor training devices like wheelchair, prone crawlers.

Walking aids: canes, crutches and walkers.

Assistive devices are useful for training functional activities for patients with limited function.

## EVIDENCE FOR PHYSICAL THERAPY IN PALLIATIVE CARE

### Need for physical therapy in palliative care

Pate *et al*,[9] and Bryan *et al*,[10] reported an earlier estimate that approximately 30% of total cancer deaths are related to poor exercise and nutrition, and when taking into consideration both cardiovascular disease and cancer, that physical inactivity contributes to as many as 250,000 premature deaths per year. Understanding the beneficial effects of exercise and physical activity, the expanding role of physical therapy in palliative care indicated a rapid growth of evidence. The physical therapist, like other members of the team, provides palliative care. Modalities range from using heat, cold, and TENS for alleviation of pain; teaching activities of daily living that accommodate to the strength and body mechanical capabilities of the patient; and designing exercises and positioning that will maintain functional ranges of motion.[11]

Toot[11] in addition, explained the interventions provided by physical therapists in hospice and palliative care that may be directed to three facets: (1) delivering direct patient care, (2) educating the patient-family care unit and fellow health professionals, and (3) functioning as a team member.

Laakso[12] emphasized that in palliative care, physical therapists are involved in the following four levels-Prevention; Acute and post-acute care; Institutional and community-based rehabilitation; and Symptom control. The most common currently occurring role of the physical therapist is in the hospital-based care. Montagnini *et al*,[13] said, in a hospital-based palliative care unit, physical therapists treat most common functional disabilities like deconditioning, pain, imbalance and focal weakness.

The importance of physical therapy is widely stated in the most-read textbook- *Oxford Textbook of Palliative Medicine*, as follows;

*Physiotherapy aims to “optimise the patient’s level of physical function and takes into consideration the interplay between the physical, psychological, social and vocational domains of function…… The physiotherapist understands the patients underlying pathological condition, but this is not the focus of treatment. The focus of physiotherapy intervention is, instead, the physical and functional sequelae of the disease and/or its treatment, on the patient.*”[14]

Physiotherapy aims to: maintain optimum respiratory function; maintain optimum circulatory function; prevent muscle atrophy; prevent muscle shortening; prevent joint contractures; influence pain control; optimize independence and function; and, education and participation of the carer.

The following section describes the role of physical therapy in common conditions that require palliative care.

### Palliative physical therapy in patients with cancer

Physical therapists have a very important role to play in holistic care of patients diagnosed with cancer as stated by Flomenhoft[15] and Rashleigh.[16]. Rashleigh[17] listed the therapeutic strategies employed by physical therapists in palliative oncology as ambulation and musculoskeletal therapy; neurological therapy; respiratory therapy; electrophysical agents; mechanical therapy; decongestive physiotherapy; and, education. Santiago-Palma and Payne[17] listed treatments used by physical therapists on cancer patients are therapeutic massage, therapeutic heat, therapeutic Cold, patient education- advice on activity modification, range of motion and strengthening exercise, training ambulation using assistive devices, environmental modification, energy conservation and work simplification techniques. Twycross[18] mentioned that physical treatment methods like massage, heat pads and TENS are useful for pain management in cancer patients.

Physical therapy treatment techniques have also been reported in cancer-related fatigue by Watson and Mock,[19] breast cancer,[20,21] prostate cancer[22] and breast cancer-related lymphedema,[23,24] older women with cancer,[25] cancer therapy-related hyperthermia,[26] and colorectal cancer.[27]

Narayanan and Koshy[28] emphasized the importance of group exercise therapy, energy conservation techniques and regular physical activity to be effective for cancer-related fatigue. Lyles *et al*,[29] said that aversiveness associated with cancer chemotherapy could be treated by relaxation training with guided imagery. Jacobsen *et al*,[30] performed a meta-analysis of 30 randomized controlled trials and they concluded treatment effect sizes to be in favor of non-pharmacological interventions. Of them both psychological and physical activity-based interventions were proven to be better in improving quality of life in patients with cancer-related fatigue.

### Palliative physical therapy in patients with neurodegenerative disorders

The Chartered Society of Physiotherapy (CSP)’s evidence summary emphasizes the effectiveness of physical therapy in the palliative care of older people.[32] Physical activity improved overall quality of life[33] and sense of well-being[34] in persons with normal ageing. Specialist palliative care has an established role in the management of patients with advanced progressive neurological disease. A proactive palliative care approach in patients with amyotrophic lateral sclerosis (ALS) can significantly improve their quality of life. Physiotherapy, counseling, addressing nutritional issues and regular respite can be supportive in ALS/ MND.[14]

#### Multiple sclerosis

Multiple sclerosis (MS)[34] is the most prevalent chronic disabling neurological disease among adults. Physical activity is indirectly associated with improved QoL through pathways that include fatigue, pain, social support, and self-efficacy in individuals with MS. Motl *et al*,[35] also found improvements in social support, self-efficacy and reduced functional limitations following physical activity program in MS patients. Besides drugs, physiotherapy is the mainstay in the management of spasticity in MS.[14]

#### Alzheimer’s disease

Weih *et al*,[36] performed a meta-analysis of cohort studies and they concluded that regular physical activity showed better benefits not only on the overall patients’ quality of life but also reduced the risk of development of the disease during ageing.

#### Spinal cord injury and brain injury

Ginis *et al*,[37] reported that spinal cord injury patients reported less pain, depression and stress, and increased quality of life and better physical self-concept after a program of aerobic and resistance exercise training. Also they had enhanced self-motivation as found by Latimer *et al*.[38] Katz *et al*,[39] earlier found psychological effects of exercise training in paraplegia patients, which was again confirmed by Nayak *et al*,[40] in their study on music therapy which showed significant positive effect on mood and social interaction in traumatic brain injury and stroke patients. In stroke rehabilitation, the most recent therapeutic advance is the use of motor imagery and mental practice techniques. Holmes[41] mentioned- *The use of motor imagery (MI)—presumed to be a visual and kinesthetic neural representation of the overt behavior—has relied on two major assumptions. The first assumption is that the internally generated movement patterns involve the same neuronal correlates as the overt behaviors (i.e., the two conditions are functionally equivalent). Second, it assumes that using MI will lead to cortical and subcortical neuronal modification that is of benefit to a person who has experienced a stroke.* Mental practice as an effective technique for locomotor training and rehabilitation was also employed in most neurological conditions as found in their comprehensive review by Malouin and Richards.[42]

### Palliative physical therapy in patients with respiratory diseases and critical illnesses

The role of physical therapy in palliative care of patients with respiratory disorders ranges from home-based care such as training symptom control for cough and breathlessness to providing interventions such as airway clearance techniques in the intensive and critical care units in hospital-based rehabilitation. The renowned professional body for respiratory and cardiac conditions, the American Thoracic Society[43] emphasised this role in its definition for pulmonary rehabilitation;

*Pulmonary rehabilitation is a multidisciplinary program of care for patients with chronic respiratory impairment that is individually tailored and designed to optimize physical and social performance and autonomy.* ATS has listed four essential components of pulmonary rehabilitation as; (1) exercise training (upper extremity endurance training, low extremity endurance training, strength training and respiratory muscle training), (2) education (breathing strategies, energy conservation and work simplification, end-of-life education), (3) psychosocial and behavioral intervention (coping strategies, stress management), and (4) outcome assessment. This was also mentioned by Lanken *et al*,[44] that pulmonary rehabilitation includes exercise training, psychosocial support, nutritional therapy, and self-management education, including breathing strategies, use of supplemental oxygen, pharmacologic therapy (to relieve airways obstruction), and panic control. The authors gave following suggestions in order to treat psychosocial factors: For anxiety, use relaxation techniques, distraction, activity modifications, behavior modifications, and breathing strategies. For depression, use cognitive therapy, antidepressants, or a combination of both.

The therapeutic efficacy of pulmonary rehabilitation was demonstrated convincingly in many systematic reviews and randomized controlled trials and hence physical therapy in the form of exercise training in globally accepted and widely practised position statements and treatment guidelines.

Nici *et al*,[45] mentioned that American Thoracic Society and European Respiratory Society has endorsed exercise training in their position statement as a comprehensive component of pulmonary rehabilitation to gain effective symptom control for dyspnea.

Of the respiratory disorders requiring palliative care, the most life-limiting condition is idiopathic pulmonary fibrosis. Raghu *et al*,[46] stated that idiopathic pulmonary fibrosis (IPF), a type of interstitial lung disease (ILD), is a progressive life-threatening disease that is characterized anatomically by scarring of the lungs and symptomatically by exertional dyspnea. Fan and Kozinetz[47] reported that the maximum life expectancy for a patient with IPF is 3-5 years. Pediatric ILD still has a shorter life span of about 47 months. In the absence of anti-inflammatory therapies failing to improve outcomes in these patients,[48] physical therapy treatment methods would definitely address the problems of cough and dyspnea by enhancing quality of life in those cases.

#### Cough

Keenleyside and Vora[49] recommended that chest physiotherapy together with steam inhalation can be given for sputum clearance in patients who complained of cough. Cough therapy as described by Fulton and Else[14] included forced exhalation, airways vibration, assisted coughing techniques, postural drainage.

#### Breathlessness

LeGrand[50] stated that breathing retraining such as diaphragmatic breathing or pursed lip breathing are useful in palliative management of dyspnea. In addition to the central role of opioids, the palliative approach to dyspnea is multidisciplinary, with the need for an individualized program including education, emotional support, physical therapy, and respiratory therapy. Syrett and Taylor[51] while emphasizing on a collaborative nurse-physiotherapist model in palliative care setup, referred to techniques of positioning, relaxation, breathing awareness exercises, walking and stair-climbing activities, coping and pacing, activity modification being useful in physiotherapy management of breathlessness. Vora[52] explained that non-drug measures such as lifestyle modification, stress management, breathing control and posture, and relaxation techniques are useful for control over breathlessness. Lox and Freehill[53] found pulmonary rehabilitation to improve both physiological and psychological measures of self efficacy for 6-min walking distance, dyspnea, fatigue, emotional function and overall quality of life in patients diagnosed with COPD.

Sachs and Weinberg[54] explained the use of both active and passive strategies in pulmonary rehabilitation. Active strategies like lower intensity exercise protocols, including interval training and single-leg ergometry similar to aerobic exercise, are effective in improving dyspnea and functional capacity. Passive strategies such as neuromuscular electrical stimulation have been demonstrated to improve muscle strength and mass and reduce exertional dyspnea. The authors also added that home-based, self-monitored programs compare favorably with outpatient hospital-based programs. There was increasing evidence that pulmonary rehabilitation was well tolerated and effective for patients with severe COPD, and that other diseases associated with disabling dyspnea would improve symptomatically with pulmonary rehabilitation.

Emery *et al*,[55] found exercise, education and stress management (EXESM) intervention produced better effects both on physiological functioning (pulmonary function, exercise endurance), psychological well-being (depression, anxiety, quality of life), and cognitive functioning (attention, motor speed, mental efficiency, verbal processing).

Ciesla[56] elaborated the role of chest physical therapy in intensive care units with use of. techniques like postural drainage, percussion, vibration, breathing exercises, cough stimulation techniques, limb mobilization, positioning and airway suctioning that were routinely performed in the treatment of critically or terminally ill patients in the intensive care units.

The reported benefits of formalized exercise training to an informal recreational physical activity in chest physical therapy also extended to include systemic conditions like chronic renal failure to have positive effects on quality of life by Eng and Ginis.[57] Also self- determined motivation of patients attending a cardiac rehabilitation was improved followed by a regular physical activity program as found by Russell and Bray.[58]

### Palliative physical therapy for people living with human immuno deficiency virus (HIV) or acquired immuno deficiency syndrome (AIDS)

Palliative care improved outcomes in patients living with HIV or AIDS. Home palliative care and in-patient hospice care improved a number of patient outcomes, particularly in terms of pain and symptom control, anxiety, insight and spiritual well-being. Harding *et al*,[59] also stated that palliation should be offered as a flexible, integrated approach when needed, across the range of institutional and home care settings, alongside new therapeutics. Dysfunction of the aerobic system as a major cause of physical disability in HIV patients was found by Cade *et al*,[60] and hence the other authors Nixon *et al*,[61] 2005, O’Brien *et al*,[62] found aerobic exercise interventions to be effective in their systematic reviews of randomized controlled trials.

Earlier proponents like O’Brien *et al*,[63] and O’Brien *et al*,[64] advocated positive effects for progressive resisted exercise in their systematic reviews. While therapeutic massage was found to be effective by Hillier *et al*,[65] in another systematic review, Crepaz *et al*,[66] found cognitive-behavioral interventions to have positive effect on mental functioning and also immune function in patients living with HIV.

### Palliative physical therapy for people with psychiatric disorders and altered mental states

Exercises as a treatment for altered psychological states have been through over the years grounded on the principle, “*sound mind and a sound body*” and authors like Wilfey and Kunce[67] and Tuckman and Hinkle[68] found earlier that exercise has not only physical effects but also psychological, which was found both in adults and in children respectively. Later other authors like King *et al*,[69] also found similar findings.

### Cognitive effects of exercise

Though exercise had been a part of behavioral medicine for treating altered physiological states like obesity, diabetes, cardiovascular risk modification and smoking according to Martin and Dubbert,[70] through its cognitive effects, according to Tomporowski and Ellis,[71] exercise also had the potential to address disorders like dementia, depression and altered mood states. Exercise also positively influences memory-search performance or reaction time,[72] cognitive abilities like reasoning,[73] working memory.[73]

#### Emotional effects of exercise

Exercise therapy programs such as aerobic exercise training was shown by McCann and Holmes[74] to positively influence depression, followed by Brown and Siegel,[75] who studied adolescent population and later McNeil *et al*,[76] confirmed this with added effects of exercise on anxiety. Exercise as an adjunct to enhanced imagery was studied by Schwartz and Kaloupek[77] for anxiety reduction. It was Bruning and Frew[78] who found exercise to be beneficial for stress management and Puetz *et al*,[79] found chronic exercise to influence feeling of energy and fatigue, and recently Chafin *et al*,[80] found exercise as an effective strategy for anger control. More recently, Jerstad *et al*,[81] found adolescent female population to benefit from physical activity in reducing their levels of depression since physical activity and depression was shown to have a reciprocal inverse relationship.

#### Intensity of exercise training and psychological effects

Steptoe and Cox[82] found that high-intensity exercise led to increases in tension/anxiety and fatigue, whereas positive mood changes (vigor and exhilaration) were seen following low-intensity exercise only.

#### Effects of exercise on psychiatric disorders

Tkachuk and Martin[83] in their review, concluded that exercise therapy was a viable, cost-effective treatment strategy for depression in psychiatric disorders and in chronic pain states. Less strenuous forms of regular exercise such as a physical activity like walking was shown to have larger health benefits compared to neuropsychiatric drugs. The exercise therapy was found useful for clinical depression, developmental disabilities, schizophrenia, somatoform disorders and substance-abuse disorders. It was Dubbert[84,85] who continuously reported and emphasized the potential of using exercise and physical activity in a scientific manner to promote mental health and also inspired toward continued efforts to understand the underlying biological, psychosocial, and cognitive mechanisms.

### Other related therapeutic techniques used by physical therapists in palliative care

Though theoretically not part of physical therapy, physical therapists are trained in the following techniques and they do perform these in their regular practice.

#### Relaxation

Tarler-Benlolo[86] emphasized the therapeutic role of relaxation and Halonen and Passman[87] found it an useful technique for reducing post-partum distress and Carey and Burish[88] found the same for cancer chemotherapy patients.

#### Other techniques

EMG Biofeedback,[89] exercise-related imagery,[90] music therapy,[91,92] play therapy,[93] virtual reality- Plante *et al*,[94] and exercise environment[95] was shown to influence perceived well-being where outdoor exercises energizes while indoor exercises relaxes,[96] and, Qigong exercises had positive effects on mood and anxiety.[97]

Other complementary therapies which are often included in physical therapist’s treatment armamentarium are acupuncture, aromatherapy, reflexology, relaxation and massage.[98]

Massage therapy as a complementary therapy technique for feet[99] and hands[100] is also effectively used by physical therapists in their routine plan of care.

## CONCLUSION

Physical therapy was shown to have positive influence on quality of life and perceived well-being in a wide range of patient populations requiring palliative care such as cancer, HIV, neurological disorders, cardiopulmonary conditions and mental illnesses.

The scope of physiotherapy practice is influenced by the ratio of qualified physiotherapists to the population. The number of physiotherapists per head of population in India is 1:212,000.[101] This often is an underestimated scope for a profession in a country with ever-growing demands for palliative care. This fact should give enough impetus to budding physical therapists to enter into the healing world of palliative care. The continuously growing numbers of in patients requiring palliative care in India necessitates professional involvement on the part of the physical therapists and mutual understanding from palliative care team members to bring about a policy change and to streamline implementation at ground-level.

Meier *et al*,[102] suggested three barriers for palliative medicine, which in turn can be applicable toward integration of physical therapy into palliative care such as: professional knowledge and skills in palliative care among therapists and other team members; professional and public attitudes about the goals of physical therapy; and financial and structural attributes of the health care industry.

Future studies are warranted on the following aspects;

1. Assessment of knowledge, attitudes, beliefs and experiences toward palliative care among physical therapists
2. Evolution of a palliative care training program for physical therapists.
3. Qualitative research on experiences of palliative care team members with physical therapists
4. Influence of physical therapy on patient and caregiver perceptions and quality of life in other palliative care conditions.

The authors wish to recommend three approaches to improve physical therapy in palliative care.

1. Improving professional knowledge and skills
2. Changing professional attitudes toward care at end-of-life
3. Recognizing the palliative care healthcare system in India.

“*Coming together is the beginning, keeping together is progress, working together is success*”

- Henry Ford.
